# Potential Joint Protective and Anti-Inflammatory Effects of Integrin α_v_β_3_ in IL-1β-Treated Chondrocytes Cells

**DOI:** 10.3390/biomedicines11102745

**Published:** 2023-10-10

**Authors:** Hun Hwan Kim, Se Hyo Jeong, Min Yeong Park, Pritam Bhagwan Bhosale, Abuyaseer Abusaliya, Hyun Wook Kim, Je Kyung Seong, Meejung Ahn, Kwang Il Park, Jeong Doo Heo, Young Sil Kim, Gon Sup Kim

**Affiliations:** 1 Research Institute of Life Science and College of Veterinary Medicine, Gyeongsang National University, Jinju 52828, Republic of Korea; shark159753@naver.com (H.H.K.); tpgy123@gmail.com (S.H.J.); lilie17@daum.net (M.Y.P.); shelake.pritam@gmail.com (P.B.B.); yaseerbiotech21@gmail.com (A.A.); kipark@gnu.ac.kr (K.I.P.); 2 Division of Animal Bioscience & Intergrated Biotechnology, Jinju 52725, Republic of Korea; hwkim@gnu.ac.kr; 3 Laboratory of Developmental Biology and Genomics, BK21 PLUS Program for Creative Veterinary Science Research, Research Institute for Veterinary Science, College of Veterinary Medicine, Seoul National University, Seoul 08826, Republic of Korea; snumouse@snu.ac.kr; 4 Department of Animal Science, College of Life Science, Sangji University, Wonju 26339, Republic of Korea; meeahn20@sangji.ac.kr; 5 Biological Resources Research Group, Bioenvironmental Science and Toxicology Division, Korea Institute of Toxicology Gyeongnam Branch (KIT), Jinju 52834, Republic of Korea; jdher@kitox.re.kr; 6 T-Stem Co., Ltd., Changwon 51573, Republic of Korea; askand@naver.com

**Keywords:** chondrocyte, anti-inflammation, chondrogenesis, integrin α_v_β_3_, IL-1β

## Abstract

In osteoarthritis (OA), the articular cartilage covering the articular surface of the bone wears out, exposing the subchondral bone, and the synovial membrane surrounding the joint becomes inflamed, causing pain and deformity. OA causes pain, stiffness, and swelling, and discomfort in the knee when climbing stairs is a typical symptom. Although drug development studies are conducted to treat these inflammatory joint diseases, it is difficult to find conclusive research results which could reduce inflammation and slow cartilage tear. The development of drugs to relieve inflammatory pain often utilizes inflammatory triggers. Interleukins, one of the proteins in the limelight as pro-inflammatory factors, are immune-system-stimulating factors that promote the body’s fight against harmful factors such as bacteria. In this study, inflammation was induced in Chondrocytes cells (Chon-001 cells) with IL-1β and then treated with integrin α_v_β_3_ to show anti-inflammatory and chondrogenesis effects. Integrin α_v_β_3_ was not toxic to Chon-001 cells in any concentration groups treated with or without IL-1β. COX-2 and iNOS, which are major markers of inflammation, were significantly reduced by integrin α_v_β_3_ treatment. Expressions of p-ERK, p-JNK, and p-p38 corresponding to the MAPKs signaling pathway and p-IκBα and p-p65 corresponding to the NF-κB signaling pathway were also decreased in a dose-dependent manner upon integrin α_v_β_3_ treatment, indicating that inflammation was inhibited, whereas treatment with integrin α_v_β_3_ significantly increased the expression of ALP, RUNX2, BMP2, BMP4, Aggrecan, SOX9, and COL2A1, suggesting that osteogenesis and chondrogenesis were induced. These results suggest that integrin α_v_β_3_ in-duces an anti-inflammatory effect, osteogenesis, and chondrogenesis on IL-1β-induced Chon-001 cells.

## 1. Introduction

Osteoarthritis is an articular degenerative disease that is characterized by persistent pain, inflammation of the joints, and restricted range of motion [1]. Chronic OA causes joint dysfunction and discomfort, progressively making it a significant financial burden [2]. In OA, the cartilage within a joint begins to deteriorate, and the underlying bone changes, pain, stiffness, and edema are all symptoms of OA. The weakening of cartilage can also alter its biomechanical, biochemical, and metabolic properties [3]. OA is a joint disease that is caused by the complex interaction between synovial membrane, cartilage, and subchondral bone [2] and can occur in all joint tissues, including the meniscus and infrapatellar fat pad [4]. According to current research, it is a complex disease where joint dysfunction, chondrodegeneration, and low-grade chronic inflammation all occur together [5]. Interleukin-1 (IL-1) is a pro-inflammatory cytokine that has been linked to the development of OA [6]. Interleukin-1β (IL-1β) is a key inflammatory cytokine in the etiology of OA, and IL-1β exposure can promote chondrocyte inflammation injury in vitro [7]. When chondrocytes were exposed to IL-1, it promoted the release of cyclooxygenase-2 (COX-2) and 5-lipoxygenase (5-LOX), which in turn produced prostaglandin E2 and LTB4, respectively [8]. Many studies have shown evidence that the release of COX-2 was increased by IL-1β treatment, which proves that IL-1β production and IL-1β-induced inflammatory mediators play an essential role in the pathogenesis of OA [9,10]. Various methods are being used to treat OA, and the most widely used treatment is the use of analgesics and non-steroidal anti-inflammatory drugs (NSAIDs) [11]. However, because side effects can occur, products based on extracted chondroitin sulfate (CS) and glucosamine have been developed [12], and research is being conducted to overcome these problems.

Nitric Oxide (NO) is a mediator that causes inflammation due to an excessive immune response and is also involved in joint inflammatory diseases [13]. Prostaglandin E2 (PGE2) also acts as an inflammatory mediator generated from arachidonic acid by cyclooxygenase (COX) [14]. Interleukin-1β (IL-1β) causes inflammation in joint cartilage, and research has shown that it stimulates the production of NO and PGE2, and many treatments targeting this have been reported [15].

Several osteogenic factors regulate the development and differentiation of osteoblasts, such as bone morphogenetic proteins (BMPs) and Runx2 [16]. RUNX2 is known to be involved in the proliferation and maturation of osteoblasts and chondrocytes by inducing an increase in ALP expression and increasing the expression of bone matrix protein genes [17]. Additionally, SOX9 is involved in the sequential differentiation of chondrocytes, and Aggrecan is essential for articular cartilage by carrying sulfate chondroitin and keratan chains [18,19]. COL1A1 is involved in forming collagen type 1, the most common type found in bone [20]. COL2A1 is involved in forming type II collagen, which supports the body’s joints [21]. Tumor necrosis factor (TNF), interleukin-6 (IL-6), and in particular interleukin-1 (IL-1) are the pro-inflammatory cytokines that can activate the production of inflammatory and catabolic factors, speeding up the progression of osteoarthritis [22]. Matrix-degrading proteases, such as matrix metalloproteinases (MMPs), are overproduced when articular cartilage degrades, which leads to the disruption of metabolic homeostasis [23,24]. According to some studies, COX-2 inhibitors can both promote the synthesis of collagen II and stop the release of pro-inflammatory cytokines [25,26].

Generally, inflammation is mediated by NF-кB and MAPKs signaling pathways. P65 and p50 make up the majority of the NF-кB heterodimer [27]. They translocate from the cytoplasm to the nucleus after being freed from IкB-α. IKK’s phosphorylation and degradation both contribute to the activation of NF-кB. After activation, NF-кB translocates into nuclei to promote the expression of pro-inflammatory genes like IL-1, IL-6, and TNF-α [28]. Members of the MAPK family include ERK 1/2, p38, and JNK, which regulate the transducer that transmits environmental stimuli to the nucleus [29]. JNK and p38 are most closely related to the pro-inflammatory mediators.

On the cell surface, integrins are transmembrane receptors that are functional heterodimers made up of subunits connected by non-covalent connections [30]. Integrins mediate interactions between cells and the extracellular matrix, allowing mechanical stimulus to be carried into the cell and subsequently start and control intracellular osteogenic signaling pathways [10]. Integrins have large extracellular domains that bind to extracellular matrix ligands, and they also have loosely structured cytoplasmic tails that can interact with a variety of intracellular proteins to bind to the cytoskeleton [6]. Focal adhesions and the differentiation of stem cells are influenced by the recruitment and activation of intracellular signaling proteins like vinculin and focal adhesion kinase (FAK) via the accumulation and activation of integrins [31].

In this study, the anti-inflammatory, chondrogenic, and osteogenic effects of integrin α_v_β_3_ in chondrocytes cells induced by inflammation through IL-1β were investigated. Overall, to confirm the anti-inflammatory effect of integrin α_v_β_3_, the expression of MAPK, MMP, NF-kB, and p-STAT3, which are key inflammatory factors, was confirmed. Additionally, the effects of integrin α_v_β_3_ on chondrogenesis and osteogenesis were confirmed by identifying factors related to chondrogenesis and osteogenesis. Therefore, we aimed to study whether integrin α_v_β_3_ could confer protection against OA in Chon-001 cells, and stimulated human chondrocytes in vitro with IL-1β to produce OA. The relationship between various factors inducing chondroprotective and anti-inflammatory effects was also investigated. Inflammation was induced in chondrocytes through IL-1β, and factors related to anti-inflammation, chondrogenesis, and osteogenesis were identified by processing integrin α_v_β_3_. It suggests the possibility of and can be used as basic data for the development of other OA treatments. 

## 2. Materials and Methods

### 2.1. Chemicals and Reagents

Integrin α_v_β_3_ was purchased from biotechne (Minneapolis, MN, USA)—Cat. no. 3050-AV-050. 3-(4,5-Dimethylthiazol-2-yl)-2,5-diphenyltetrazolium bromide (MTT) was purchased from Duchefa Biochemie (Haarlem, The Netherlands). Antibodies to COX-2 (cat. no. 12282S, monoclonal(M)), ERK (Extracellular-signal-regulated kinase) (cat. no. 4695S, M), phosphorylated ERK (p-ERK) (cat. no. 4370S, M), JNK (c-Jun N-terminal kinases) (cat. no. 9258S, M), p-JNK (P-c-Jun N-terminal kinases) (cat. no. 4671S, M), p38 (cat. no. 8690S, M), p-p38 (cat. no. 9216S, M), STAT3 (Signal transducer and activator of transcription 3) (cat. no. 4904S, M), p-STAT3 (P-Signal transducer and activator of transcription 3) (cat. no. ab30647, M), ALP (Alkaline phosphatase) (cat. no. ab229126, M), RUNX2 (RUNX Family Transcription Factor 2) (cat. no. 12556S, M), BMP2 (bone morphogenetic protein 2) (cat. no. ab214821, M), BMP4 (cat. no. ab39973, M), IκBα (cat. no. 4812S, M), p-IκBα (cat. no. 14D4, M), p65 (cat.no. 8242S, M), p-p65 (cat. no. 3033S, M), MMP-2 (cat.no. ab97779, M), MMP-9 (cat. no. BS1241, M), COL1A1 (cat.no. 39952S, M) COL2A1 (cat.no. 43306S, M), Sox9 (cat.no.82630S, M), Aggrecan (cat. no. 28971S, M), and β-actin (cat. no. 4970S, M) were purchased from Cell Signaling Technology (Danvers, MA, USA). Horseradish peroxidase (HRP)-conjugated secondary antibodies to anti-rabbit (cat. no. A120-101P) and anti-mouse (cat. no. A90-116P) were obtained from Bethyl Laboratories, Inc. (Montgomery, AL, USA).

### 2.2. Cell Culture and Integrin Treatment

The American Type Culture Collection (ATCC, University Boulevard Manassas, Manassas, VA, USA) provided the human chondrocytes Chon-001 cells, which were grown in full DMEM with 10% heat-inactivated fetal bovine serum (FBS), 100 U/mL penicillin, and 100 g/mL streptomycin. The cells were incubated at a temperature of 37 °C in a humid atmosphere with 5% CO_2_. After seeding the cells, integrin α_v_β_3_ was added at a concentration of 25 and 50 ng/mL with IL-1β, and it spent 24 h incubating in an incubator.

### 2.3. Cell Viability Assay

Chon-001 cells(#3) were seeded at a density of 1 × 10^4^ cells per well in 96-well plates and then cultured, with IL-1β (10 ng/mL) and co-treatment with various concentrations of integrin α_v_β_3_ (0, 0.5, 1, 2, 5, 10, 25, 50, and 100 ng/mL) at 37 °C for 24 h. MTT solution (10 µL; 5 mg/mL) was applied to the plate and allowed to incubate for 2 h at 37 °C. The growing medium was completely removed, followed by the dissolution of the insoluble formazan crystals in DMSO. Microplate reader Multiskan FC (Thermo Scientific, Rockford, IL, USA) was used to measure the absorbance of the converted dye at a wavelength of 560 nm.

### 2.4. Nitric Oxide (NO) Assay

Chon-001 cells(#3) were seeded at 1 × 10^4^ cells, treated with or without IL-1β (10 ng/mL), and cultured at 37 degrees for 1 h. Subsequently, the cells were treated with integrin α_v_β_3_ (0, 0.5, 1, 2, 5, 10, 25, 50, and 100 ng/mL) and cultured for 24 h. In total, 100 ul of supernatant was analyzed with a NO Plus detection kit (iNtRON Biotechnology, Gyeonggi, Republic of Korea); Cat. No. 21023). According to the manufacturer’s instructions, 50 ul of N1 and N2 buffers were added and measured at 520 nm with a Microplate reader Multiskan FC (Thermo Scientific, Rockford, IL, USA). The NO concentration of each group used a standard, and the curve was prepared using standard sodium nitrite.

### 2.5. Prostaglandin E2 (PGE2) Enzyme-Linked Immunoassay (ELISA)

Chon-001 cells(#4) were seeded at 5 × 10^4^ cells in a 24-well plate, pre-treated with or without IL-1β (10 ng/mL), and cultured at 37 degrees for 1 h. After that, the cells were treated with integrin α_v_β_3_ (25 and 50 ng/mL) and cultured for 24 h. In total, 100 μL of supernatant was used for the PGE2 measurement. PGE2 levels were measured using the PGE2 ELISA kit (Cat. No. ADI-900-001; Enzo Life Sciences, Inc., New York, NY, USA) according to the product instructions. The data were handled with an immunoassay utilizing a 4-parameter logistic curve fitting program.

### 2.6. Western Blot

For Western blot analysis, Chon-001 cells(#4~#12) were seeded into 60 mm plates at a density of 1 × 10^6^ cells/well and treated with 25 and 50 ng/mL integrin α_v_β_3_ for 24 h at 37 °C. The cells that had been treated were then lysed using a radio immune precipitation assay (RIPA) buffer from iNtRON Biotechnology in Gyeonggi, Republic of Korea, which comprises a protease inhibitor cocktail and a phosphatase inhibitor from Thermo Fisher Scientific in Waltham, MA, USA. Protein concentrations were measured using the Pierce TM BCA Protein Assay (Thermo Scientific, Rockford, IL, USA) in accordance with the manufacturer’s instructions. The JP/WSE-4040 HorizeBLOT 4M-R WSE-4045 (ATTO Blotting System, ATTO Technology, New York, NY, USA) was used to transfer equal quantities of protein (10 μg) onto PVDF membranes after SDS-PAGE separation on 10% gels.

EzBlockChemi (ATTO Technology, New York, USA) was then used to block the blots for 1 h while they were at room temperature. Additional primary antibody dilutions (dissolved in EzBlockChemi (ATTO Technology, New York, NY, USA) were incubated on the membranes for an additional overnight period at 4 °C. The membranes were TBS-T washed 5 times for 10 min before being probed for 2 h at room temperature with a second antibody. A 1:5000 dilution (dissolved in EzBlockChemi) of the second antibody was used. With β-actin serving as the loading reference, the blots were quantified via densitometry using Image J software (https://imagej.nih.gov) from the National Institutes of Health. The blots were visualized using the Clarity™ ECL substrate reagent from Bio Rad Laboratories, Inc., Hercules, CA, USA

### 2.7. Statistical Analysis

All the experimental data were expressed as the mean ± SEM. One-way factorial analysis of variance (ANOVA) followed by Dunnett’s multiple comparisons test was performed using GraphPad Prism program (version 9.3.1 for window, GraphPad Software, Boston, MA, USA, www.graphpad.com, accessed on 5 June 2023). The *p*-value of <0.05 was considered statistically significant.

## 3. Results

### 3.1. The Effect of Integrin α_v_β_3_ on Chon-001 Cell

**Figure 1:**
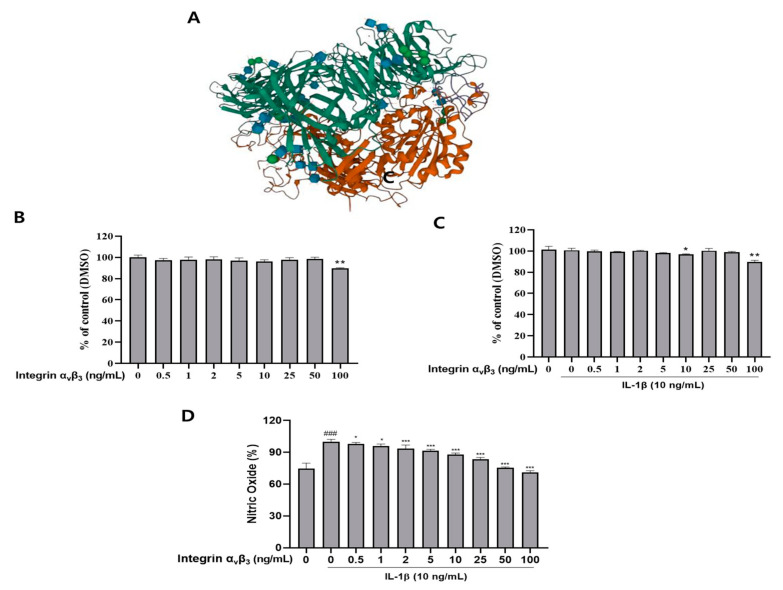
Cytotoxic effect and NO assay of integrin α_v_β_3_ treated with or without IL-1β (10 ng/mL) and treated with integrin α_v_β_3_ (0, 0.5, 1, 2, 5, 10, 25, 50, and 100 ng/mL) on Chon-001 cells at 37 °C for 24 h. (**A**) Protein structure of integrin α_v_β_3_ (RCSB PDB (https://www.rcsb.org, accessed on 15 June 2023), ID 1JV2). (**B**) Integrin α_v_β_3_ (0, 0.5, 1, 2, 5, 10, 25, 50, and 100 ng/mL) was treated by concentration without IL-1β, and further toxicity of integrin α_v_β_3_ to cell for 24 h was measured. (**C**) Integrin α_v_β_3_ treated with (0, 0.5, 1, 2, 5, 10, 25, 50, and 100 ng/mL) concentrations with IL-1β. (**D**) Effect of integrin α_v_β_3_ on IL-1β-induced NO release from Chon-001 cells. Cell viability when IL-1β and integrin α_v_β_3_ were treated together for 24 h. ^###^ *p* < 0.001 vs. untreated group; * *p* < 0.05, ** *p* < 0.01, *** *p* < 0.001 vs IL-1β treated group.

The structure of integrin α_v_β_3_ is shown in Figure 1A. An MTT assay was carried out to investigate the cytotoxic effect of integrin α_v_β_3_ on Chon-001 cells. We incubated Chon-001 cells with various concentrations (0, 0.5, 1, 2, 5, 10, 25, 50, and 100 ng/mL) of integrin α_v_β_3_ for 24 h with and without IL-1β (10 ng/mL), and cytotoxicity was determined. As shown in Figure 1C, all concentration groups that treated integrin α_v_β_3_ with IL-1β (10 ng/mL) showed more than 80% cell survival. We followed a nontoxic concentration of integrin α_v_β_3_ (25 and 50 ng/mL) for subsequent experiments to rule out possible mechanisms (Figure 1B,C).

#### NO Inhibition of Integrin α_v_β_3_ in IL-1β-Induced Chon-001 Cells

As a result of treating integrin α_v_β_3_ in IL-1β-induced Chon-001 cells, it can be confirmed that NO production is suppressed (Figure 1D). These results showed that NO was significantly increased when Chon-001 cells were treated with IL-1β, and NO was significantly decreased in a dose-dependent manner when treated with integrin α_v_β_3_. Therefore, integrin α_v_β_3_ downregulates NO, which plays an important role in mediating inflammation and suppresses the initial inflammatory response.

### 3.2. Effects of Integrin α_v_β_3_ on COX-2 Expression of IL-1β-Induced Chon-001 Cells

**Figure 2:**
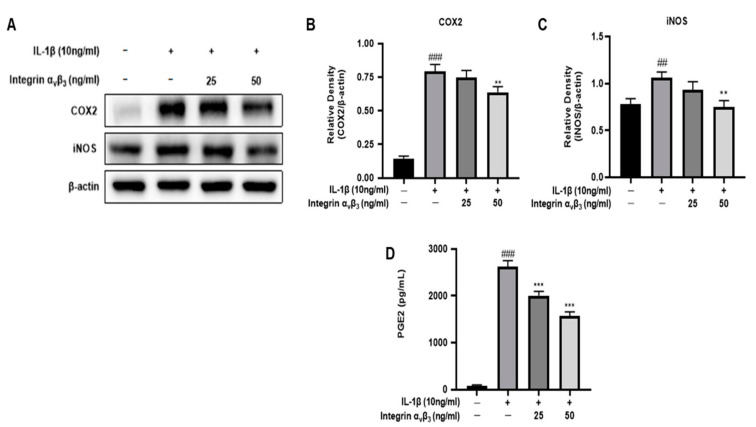
The anti-inflammatory effect with or without integrin α_v_β_3_ in IL-1β-induced Chon-001 cells. The relative density of COX-2 and iNOS expression decreased in a dose-dependent manner. (**A**) The expression of COX2 and iNOS were quantified via Western blot analysis. (**B**) Expression of COX2 with or without IL-1β (10 ng/mL). (**C**) Expression of iNOS with or without IL-1β (10 ng/mL). (**D**) Effect of integrin α_v_β_3_ on IL-1β-induced PGE2 release from Chon-001 cells. Comparison with only IL-1β ** *p* < 0.01, *** *p* < 0.001. Comparison with integrin α_v_β_3_ and IL-1β-treated group ^##^ *p* < 0.01, ^###^ *p* < 0.001.

Several stimuli induce COX-2 and are responsible for the production of large amounts of pro-inflammatory cytokines [32,33]. As shown in Figure 2, COX-2 and iNOS expressions were markedly upregulated in response to IL-1β; however, the co-treated group showed downregulation in a concentration-dependent manner. This result suggests that integrin α_v_β_3_ could inhibit the COX-2 and iNOS expression level in Chon-001 cells.

#### Effect of Integrin α_v_β_3_ on PGE2 Expression in Chon-001 Cells Induced by IL-1β

Normal Chon-001 cells were treated with IL-1β to induce an increase in PGE2 and then treated with integrin α_v_β_3_ in a dose-dependent manner, resulting in a significant decrease in PGE2 (Figure 2D). In Figure 2A,B, COX2 was significantly reduced due to integrin α_v_β_3_, which predicted a decrease in PGE2. In Figure 2D, a decrease in PGE2, an inflammatory mediator, was also confirmed.

### 3.3. Effect of MAPK Pathway by Integrin α_v_β_3_

**Figure 3:**
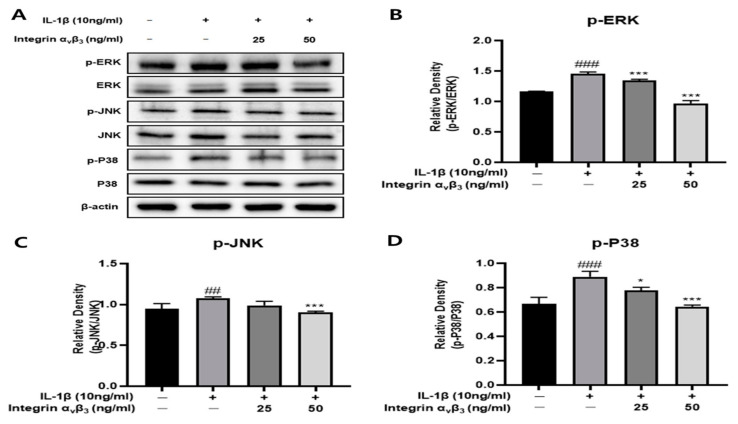
Effect of integrin α_v_β_3_ with or without IL-1β-induced MAPKs protein expression in Chon-001 cells. (**A**) The expression of MAPKs was quantified via Western blot analysis. (**B**) Expression of p-ERK affected by integrin α_v_β_3_. (**C**) Expression of p-JNK affected by integrin α_v_β_3_. (**D**) Expression of p-p38 affected by integrin α_v_β_3_. Comparison with only IL-1β * *p* < 0.05, *** *p* < 0.001. Comparison with integrin α_v_β_3_ and IL-1β-treated group ^##^ *p* < 0.01, ^###^ *p* < 0.001.

MAPKs (JNK, ERK, and p38) are normally not phosphorylated and exist in the cytoplasm, but are phosphorylated by stimulation of IL-1β, penetrate into the nucleus, and are involved in inducing inflammation. As shown in Figure 3, when Chon-001 cells were treated with IL-1β, the phosphorylation of JNK, p38, and ERK was significantly increased compared to the control group. However, integrin α_v_β_3_ suppressed their expression in a concentration-dependent manner. These results indicate that integrin exerts an anti-inflammatory effect by suppressing the regulation of the MAPK signaling pathway in Chon-001 cells induced by IL-1β inflammation.

### 3.4. Inhibition of IL-1β-Induced NF-κB Pathway Activation by Integrin α_v_β_3_

**Figure 4:**
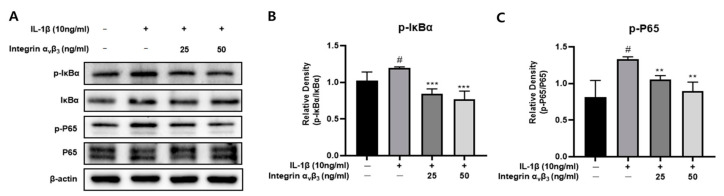
Effect of integrin α_v_β_3_ with or without IL-1β-induced protein expression of p-p65 and p-IκBα in Chon-001 cells. Chon-001 cells were treated with integrin α_v_β_3_ (0, 25, and 50 ng/mL) with or without IL-1β (10 ng/mL) at 37 °C 24 h. (**A**) The expression of NF-κB was quantified via Western blot analysis. (**B**) Relative density of p-IκBα for IκBα. (**C**) Relative density of p-P65 for P65. Comparison with only IL-1β ** *p* < 0.01, *** *p* < 0.001. Comparison with integrin α_v_β_3_ and IL-1β-treated group ^#^ *p* < 0.05.

Phosphorylation and degradation of IκBα are essential for NF-κB activation, resulting in inflammation. The anti-inflammatory effect of integrin α_v_β_3_ on the NF-κB signaling pathway in IL-1β-induced Chon-001 cells was investigated. As shown in Figure 4, it was confirmed that the expression of p-IκBα and p-p65 significantly increased in Chon-001 cells in which inflammation was induced by IL-1β. However, it was confirmed that the expression of p-IκBα and p-p65 decreased in a concentration-dependent manner by integrin α_v_β_3_ treatment. These results suggest that integrin α_v_β_3_ may have an anti-inflammatory effect by suppressing the expression of p-IκBα and p-p65, which are pro-inflammatory factors involved in the NF-κB signaling pathway.

### 3.5. Effect of Integrin α_v_β_3_ on STAT3

**Figure 5:**
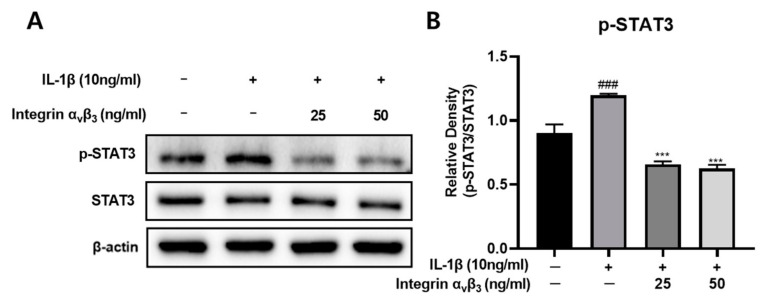
Effect of integrin α_v_β_3_ with or without IL-1β-induced protein expression of p-STAT3 in Chon-001 cells. (**A**) The expression of p-STAT3 was quantified via Western blot analysis. (**B**) Relative density of p-STAT3 for β-actin. Comparison with only IL-1β *** *p* < 0.001. Comparison with integrin α_v_β_3_ and IL-1β-treated group ^###^ *p* < 0.001.

It has been reported that STAT3, which is closely related to the induction of inflammation, is an important factor in the pathogenesis of OA. Therefore, the inhibitory effect of cartilage inflammation was confirmed by confirming the STAT3 inhibitory effect of integrin in Chon-001 cells induced by IL-1β inflammation. As shown in Figure 5, treatment with IL-1β significantly increased the expression of p-STAT3 in Chon-001 cells. However, treatment with integrin suppressed the expression of p-STAT3 in a concentration-dependent manner. This suggests that it suppresses inflammation by suppressing the expression of STAT3, which acts as an important factor in the pathogenesis of OA.

### 3.6. Effect of Integrin α_v_β_3_ on Osteogenesis

**Figure 6:**
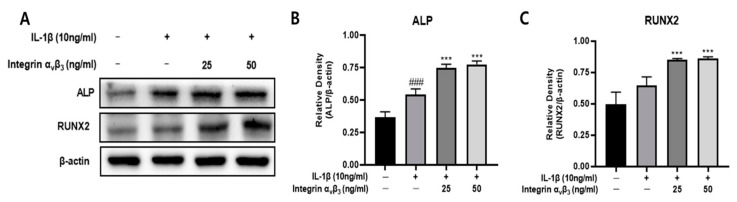
Effect of integrin α_v_β_3_ with or without IL-1β-induced protein expression of ALP and RUNX2 in Chon-001 cells. (**A**) The expression of ALP and RUNX2 were quantified via Western blot analysis. (**B**) Relative density of ALP for β-actin. (**C**) Relative density of RUNX2 for β-actin. Comparison with only IL-1β *** *p* < 0.001. Comparison with integrin α_v_β_3_ and IL-1β-treated group ^###^ *p* < 0.001.

ALP and RUNX2 are both osteoblastic markers [24]. These markers are known to be expressed by terminal hypertrophic chondrocytes [34]. ALP and RUNX2 are involved in bone formation, and integrin α_v_β_3_ alleviates osteo-inflammation [35]. Therefore, Western blots showed that protein expression of ALP and RUNX2 increased upon upregulation of integrin α_v_β_3_ expression (Figure 6). These results suggest that integrin α_v_β_3_ promotes the expression of osteogenic factors in osteogenesis.

### 3.7. Effect of Integrin α_v_β_3_ on Chondrogenesis

**Figure 7:**
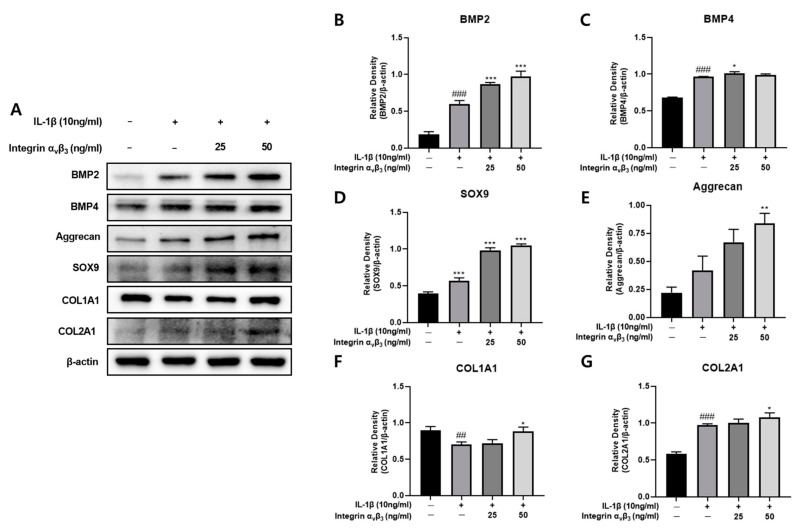
Effect of integrin α_v_β_3_ with or without IL-1β-induced protein expression of BMP2 and BMP4 in Chon-001 cells. (**A**) The expression of BMP2, BMP4, SOX9, COL1A1, COL2A1, and Aggrecan were quantified via Western blot analysis. (**B**) Relative density of BMP2 for β-actin. (**C**) Relative density of BMP4 for β-actin. (**D**) Relative density of SOX9 for β-actin. (**E**) Relative density of Aggrecan for β-actin. (**F**) Relative density of COL1A1 for β-actin. (**G**) Relative density of COL2A1 for β-actin. Comparison with only IL-1β * *p* < 0.05, ** *p* < 0.01, *** *p* < 0.001. Comparison with integrin α_v_β_3_ and IL-1β-treated group ^##^ *p* < 0.01, ^###^ *p* < 0.001.

Chondrogenesis is the earliest phase of skeletal development [35]. This includes condensation of progenitors, chondrocyte differentiation, and deposition of the chondrocyte extracellular matrix (ECM). During chondrogenesis, BMPs lead to the formation of cartilage and bone [36]. Additionally, SOX9 is an essential transcription factor in chondrogenesis and developing cartilage [37]. Among the two types of collagen (type I and II), type II collagen plays an essential role in joint health. COL2A1 also leads to the construction of type II collagen, which plays a vital role in the normal embryonic development of the skeleton as well as the capacity of cartilage [38]. Treating osteoblasts with integrin α_v_β_3_ increases protein expression of BMP2, BMP4, SOX9, and COL2A1 in a concentration-dependent manner (Figure 7). On the other hand, COL1A1 did not show a significant increase. This result suggests that integrin α_v_β_3_ promotes the expression of chondrogenesis factors.

In addition to osteogenesis and chondrogenesis, many factors affect the skeletal system. Aggrecan makes up the main structure of cartilage and maintains the structure and function of cartilage [39]. As shown in Figure 7, when Chon-001 cells were treated with IL-1β in a dose-dependent manner, the expression of Aggrecan was confirmed to increase significantly.

### 3.8. Effect of MMP on the Processing of Integrin α_v_β_3_

**Figure 8:**
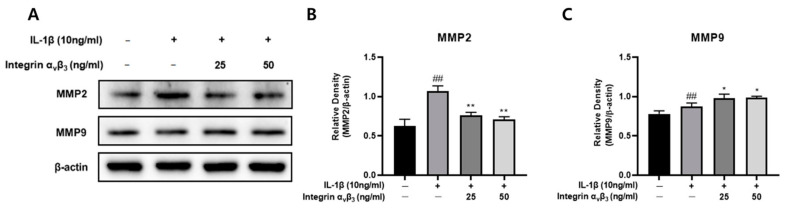
Effect of integrin α_v_β_3_ with or without IL-1β-induced protein expression of MMP2 and MMP9 in Chon-001 cells. (**A**) The expression of MMP2 and MMP9 was quantified via Western blot. (**B**) A significant decrease in MMP2 from 25 and 50 ng/mL. (**C**) In MMP9, there was a similar protein expression level. Comparison with only IL-1β * *p* < 0.05, ** *p* < 0.01. Comparison with integrin α_v_β_3_ and IL-1β-treated group ^##^ *p* < 0.01.

MMP2 and MMP9 are matrix-degrading proteases that, when overproduced, lead to joint breakdown. This reduction in MMPs may protect cartilage from damage. As shown in Figure 8, it was confirmed that the expression of MMP2 significantly increased when Chon-001 cells were treated with IL-1β, but significantly decreased when treated with integrin α_v_β_3_. However, it was confirmed that there was no significant difference in MMP9. Our results showed that integrin α_v_β_3_ reduced the expression of MMP2 induced by IL-1B, suggesting that integrin α_v_β_3_ suppresses the expression of inflammation.

## 4. Discussion

The most common degenerative joint disease, OA, causes discomfort and a loss of joint functionality. Although artificial joint replacement is always required in end-stage disease, a drug intervention is one of the most popular options for treating OA. NSAIDs and COX-2 inhibitors were widely utilized in OA treatment to reduce chronic pain and edema [40,41,42]. Therefore, research into new potent molecules for treating OA is necessary.

The primary cytokine in the pathophysiology of OA is thought to be IL-1β, which might increase the expression of pro-inflammatory cytokines and accelerate the development of OA [43]. The pathogenesis of OA is influenced by the overproduction of pro-inflammatory cytokines such as tumor necrosis factor (TNF) and IL-1β, which upregulate MMPs, and the upregulation of MMPs first induces extracellular matrix degradation, leading to cellular causes of apoptosis [44]. Previous studies have reported that IL-1β induces inflammation by activating nuclear factor (NF)-κB and mitogen-activated protein kinase (MAPK). Proteins involved in both signaling pathways not only regulate the expression of several inflammatory cytokines, but also regulate the expression of proteases, mediating various mechanisms including the inflammatory response in chondrocytes [45]. 

Among OA, the part that causes inflammation is related to COX-2 [46]. COX-2 expression alters extracellular matrix structure and function [47]. The role of the COX-2 pathway in creating an immunosuppressive microenvironment and in initiation was discussed [48]. In chondrocytes treated with integrin α_v_β_3_, COX-2 was decreased in a concentration-dependent manner (Figure 2). As an additional experiment, NO and PGE2 were confirmed by treating Chon-001 cells inflamed by IL-1β with integrin α_v_β_3_. As a result, these inflammatory mediators showed a significant decrease (Figure 1D and Figure 2D). Thus, we demonstrated that integrin α_v_β_3_ can also reduce inflammation.

One of the crucial mechanisms in eukaryotic cells that regulates and controls the structure and function of the cell is the mitogen-activated protein kinase (MAPK) signal. With more comprehensive investigation, studies discovered a strong correlation between osteoarthritis cartilage injury and the activation of the p38, ERK, and JNK signal pathways [49]. Similarly, p-JNK, p-ERK, and p-p38 all experience concentration-dependent declines after being treated with integrin α_v_β_3_. This suggests that integrin α_v_β_3_ plays a role in preventing OA by inducing the MAPK pathway (Figure 3).

NF-κB is a crucial transcriptional regulator of inflammatory factors and is essential to the pathophysiology of OA. Pro-inflammatory cytokines, matrix-degrading enzymes, and other substances can activate the NF-κB pathway. The members of NF-kB, p-IkBa, and p-P65 decrease when medicated (Figure 4). Phosphorylation of p65 causes it to be swiftly translocated to the nucleus, which increases the production of genes linked to OA, including IL-1, IL-6, and MMPs [50]. It can be seen that this drug works less to cause OA.

OA is also linked to the STAT3 pathway. During the evolution of OA, inflammatory cytokines including IL-6 and TNF-α control the activation of the STAT3 pathway. Previous research has shown that osteoarthritis models have much greater levels of STAT3 activation in articular cartilage [51]. Inhibiting the STAT3 pathway may lessen apoptosis in chondrocytes that have undergone damage and delay or stop the progression of OA (Figure 5). Integrin α_v_β_3_ reduced the STAT3 protein. It can be seen that STAT3 is inhibited by integrin α_v_β_3_. This can delay the lifespan of cartilage in bone cells.

RUNX2 is a transcription factor belonging to the RNUX family [52], and it has been reported that mice deficient in RUNX-2 lack osteoblasts and completely lack bone formation [53]. In addition, RUNX2 is essential for chondrocyte maturation by upregulating the expression of Ihh (Indian hedgehog) [54], and is known to induce the activity of alkaline phosphatase (ALP) [55]. Ihh is an essential factor for differentiation from perichondrocytes by regulating the proliferation and maturation of chondrocytes [56]. Therefore, it is suggested that the increase in RUNX2 can induce the differentiation of chondrocytes by inducing the expression of Ihh. Alkaline phosphatase (ALP), located on the cell surface of chondrocytes, hydrolyzes pyrophosphate into inorganic phosphate and is known to be involved in the proliferation and maturation of chondrocytes [17,57]. It has been reported that RUNX2 induces the activity of ALP, induces the expression of bone matrix protein genes, and induces osteoblast mineralization [58]. In conclusion, it is suggested that increased expression of RUNX2 induces the proliferation and differentiation of chondrocytes by upregulating ALP and Ihh, thereby increasing chondrogenesis. As shown in Figure 6, it was confirmed that the expression of ALP and RUNX2 significantly increased when integrin α_v_β_3_ was treated with chondrocyte Chon-001 cells. It is believed that integrin α_v_β_3_ induces differentiation and proliferation of chondrocyte Chon-001 cells by inducing the activities of ALP and Ihh through the expression of RUNX2.

BMP2 (bone morphogenetic protein 2) and bone morphogenetic protein 4 (BMP4) are reported to be involved in cartilage formation as part of the HH pathway [59]. To confirm the effect of integrin on chondrogenesis, changes in the expression of BMP2 and BMP4 in Chon-001 were confirmed (Figure 7A). When Chon-001 cells were treated with integrin α_v_β_3_, the expression of BMP2 and BMP4 significantly increased, confirming that integrin induces a positive effect on cartilage regeneration. SOX9 plays a key role in the differentiation of chondrocytes, and COL2A1 is a major component of the cartilage matrix [18,21]. Also, when Chon-001 cells were treated with integrin α_v_β_3_, a significant increase in SOX9 and COL2A1 was confirmed (Figure 7G). COL1A1 decreased but then recovered to the control level (Figure 7F). Aggrecan was also confirmed to have significantly increased, ensuring that integrin α_v_β_3_ also affects the maintenance of cartilage structure and function (Figure 7E). Therefore, integrin α_v_β_3_ can be seen to have a significant effect in controlling cartilage formation and differentiation in Chon-001 cells.

In both healthy physiological processes including embryonic development, reproduction, and tissue remodeling and in pathological processes like arthritis and metastasis, proteins of the matrix metalloproteinase (MMP) family are involved in the destruction of the extracellular matrix [60]. MMP proteins obliterate the extracellular matrix in pathological processes like metastasis and arthritis. Due to its propensity to suppress MMP, it does not eradicate processes like arthritis. In our research, MMPs often decreased (Figure 8).

**Figure 9:**
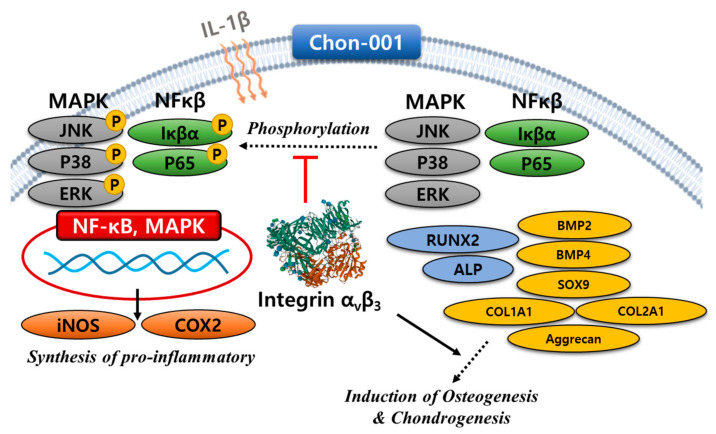
Schematic representation of anti-inflammation, osteogenesis, and chondrogenesis by integrin α_v_β_3_.

Overall, in the inflammation induced by IL-1β in Chon-001 cells, integrin α_v_β_3_ suppresses inflammation through the regulation of mitogen-activated protein kinase (MAPK) and NF-kB factors and induces factors related to osteogenesis and chondrogenesis, as shown in Figure 9.

In this study, inflammation was induced in Chon-001 cells, which are chondrocytes, through IL-1β, and integrin was treated to investigate the expression of factors related to anti-inflammation, osteogenesis, and chondrogenesis. As a result, integrin α_v_β_3_ suppressed inflammatory factors and increased the expression of chondrogenic and osteogenesis-related factors. This not only proves the possibility of integrin α_v_β_3_ as a candidate substance for treating arthritis but could also be used as basic data for the development of a therapeutic agent for arthritis. Our study has a limitation in that there are no results on primary cells and animal experiments, but we plan to improve this in future research.

## Data Availability

The data used to support the findings of this study are available upon request from the corresponding author.
